# Effects of forest types on leaf functional traits and their interrelationships of *Pinus massoniana* coniferous and broad‐leaved mixed forests in the subtropical mountain, Southeastern China

**DOI:** 10.1002/ece3.5259

**Published:** 2019-05-22

**Authors:** Juan Qin, Zhouping Shangguan

**Affiliations:** ^1^ School of Resources and Environment Anhui Agricultural University Hefei China; ^2^ State Key Laboratory of Soil Erosion and Dryland Farming on the Loess Plateau Northwest A&F University Yangling China

**Keywords:** coniferous and broad‐leaved mixed forests, forest types, leaf functional traits, *Pinus massoniana*, subtropical mountain

## Abstract

Leaf functional traits are widely used to detect and explain adaptations that enable plants to live under various environmental conditions. This study aims to determine the difference in leaf functional traits among four forest types of *Pinus massoniana* coniferous and broad‐leaved mixed forests by leaf morphological, nutrients, and stoichiometric traits in the subtropical mountain, Southeastern China. Our study indicated that the evergreen conifer species of *P. massoniana* had higher leaf dry matter content (LDMC), leaf C content, C/N and C/P ratios, while the three deciduous broad‐leaved species of *L. formosana*, *Q. tissima*, and *P. strobilacea* had higher specific leaf area (SLA), leaf N, leaf P nutrient contents, and N/P ratio in the three mixed forest types. The results showed that the species of *P. massoniana* has adapted to the nutrient‐poor environment by increasing their leaf dry matter for higher construction costs thereby reducing water loss and reflects a resource conservation strategy. In contrast, the three species of *L. formosana*, *Q. tissima*, and *P. strobilacea* exhibited an optimized resource acquisition strategy rather than resource conservation strategy in the subtropical mountain of southeastern China. Regarding the four forest types, the three mixed forest types displayed increased plant leaf nutrient contents when compared to the pure *P. massoniana* forest, especially the *P. massoniana*–*L. formosana* mixed forest type (PLM). Overall, variation in leaf functional traits among different forest types may play an adaptive role in the successful survival of plants under diverse environments because leaf functional traits can lead to significant effects on leaf function, especially for their acquisition of nutrients and use of light. The results of this study are beneficial to reveal the changes in plant leaf functional traits at the regional scale, which will provide a foundation for predicting changes in leaf traits and adaptation in the future environment.

## 1 INTRODUCTION

Generally, leaves are exposed and sensitive to external environment, thus, the response of leaf functional traits to the changes in environmental factors can enable plants to adapt to diversified habitats and thereby represent the successful ecological strategy of plants (Cochrane, Hoyle, Yates, Neeman, & Nicotra, 2016; Poorter, Niinemets, Poorter, Wright, & Villar, 2009; Xiao, Wang, Liu, Wang, & Du, 2015). Recent analyses of the significance of selected leaf functional traits have considered mostly “soft” traits because these are morphological or behavioral and are easily measured across a large number of species and sites, such as leaf size, leaf area, leaf dry mass, leaf dry matter content, and specific leaf area (Lavorel et al., 2007; Reich, Wright, & Lusk, 2007). These “soft” leaf traits can indicate how plants acquire and use resources in the diversified habitats and be used to infer adaptation in a selective context, and thus, plant defenses can be approached as multiple attributes (e.g., defense syndromes) that interact synergistically to maximize plant fitness (Agrawal & Fishbein, 2006).

Specific leaf area (SLA) is known to be a good indicator of the trade‐off between resource capture and conservation of plant species. It also links plant carbon and water cycles and provides information on the spatial variation of photosynthetic capacity and leaf nitrogen content (Pierce, Running, & Walker, 1994). Usually, leaves with high SLA values have high resource acquisition and growth rates, high water and nutrient availability, and low leaf construction investments, whereas leaves with low SLA values tend to invest more on leaf construction investments and have relatively low growth rates and nutrient availability (Pietsch et al., 2014; Wright et al., 2007). Leaf dry matter content (LDMC) reflects plant growth rate and carbon assimilation and also is a better predictor of location on an axis of resource capture, usage, and availability (Wilson, Thompson, & Hodgson, 1999). Meanwhile, leaf nitrogen (N) and phosphorus (P) concentrations, and leaf thickness (LT) are essential indices of leaf functional traits because these indices can also characterize the resource‐use strategy and photosynthetic capacity of plant species effectively and strongly correlated with SLA and LDMC (Reich et al., 1999; Wang, Zhou, Jiang, & Liu, 2017).

In recent years, many studies attempted using leaf functional traits to predict plant responses in different ecosystems, such as tropical dry forest ecosystems (Silva, Espírito‐Santo, & Morais, 2015); subtropical forest ecosystems (Costa‐Saura, Martínez‐Vilalta, Trabucco, Spano, & Mereu, 2016; Valera‐Burgos, Zunzunegui, & Díaz‐Barradas, 2013); temperate forest ecosystems (Ali et al., 2016; Li, Pei, Kéry, Niklaus, & Schmid, 2017); and desert steppe (Liu, Zeng, Lee, Fan, & Zhong, 2008). Through these studies, we can conclude that leaf trait variation is very closely related to their functional adaptation strategies of species that allow them to perform under the environmental conditions prevailing in their habitat. Furthermore, multiple leaf functional traits do not vary independently in integrated phenotype but form patterns of coordination and this coordination of traits can emerge from many sources, including developmental (e.g., allometric) constraints, genetic constraints, and resource investment trade‐offs (Li et al., 2017). It is to be expected that leaf functional traits reflect many ecosystem processes. Particularly changes in leaf economics spectrum are expected to track environmental transformation in different district.

The objective of this study was to determine the differences in leaf functional traits and the relationship among the leaf traits for four *Pinus massoniana* forest types (the pure *Pinus massoniana* forest [PF], the *Pinus massoniana*–*Quercusacu tissima* mixed forest [PQM], the *Pinus massoniana*–*Liquidambar formosana* mixed forest [PLM], and the *Pinus massoniana*–*Platycarya strobilacea* mixed forest [PPM]) of the typical forest species in the subtropical mountain of Southeastern China. Specifically, the following research questions were addressed: (a) How do leaf functional traits of the four species vary with forest types? (b) What are the correlations between leaf morphological, nutrients, and stoichiometric traits among the four forest types. And (c) What are the different resource utilization strategies of the four tree species in different forest types?

## 2 MATERIALS AND METHODS

### 2.1 Study area

**Figure 1:**
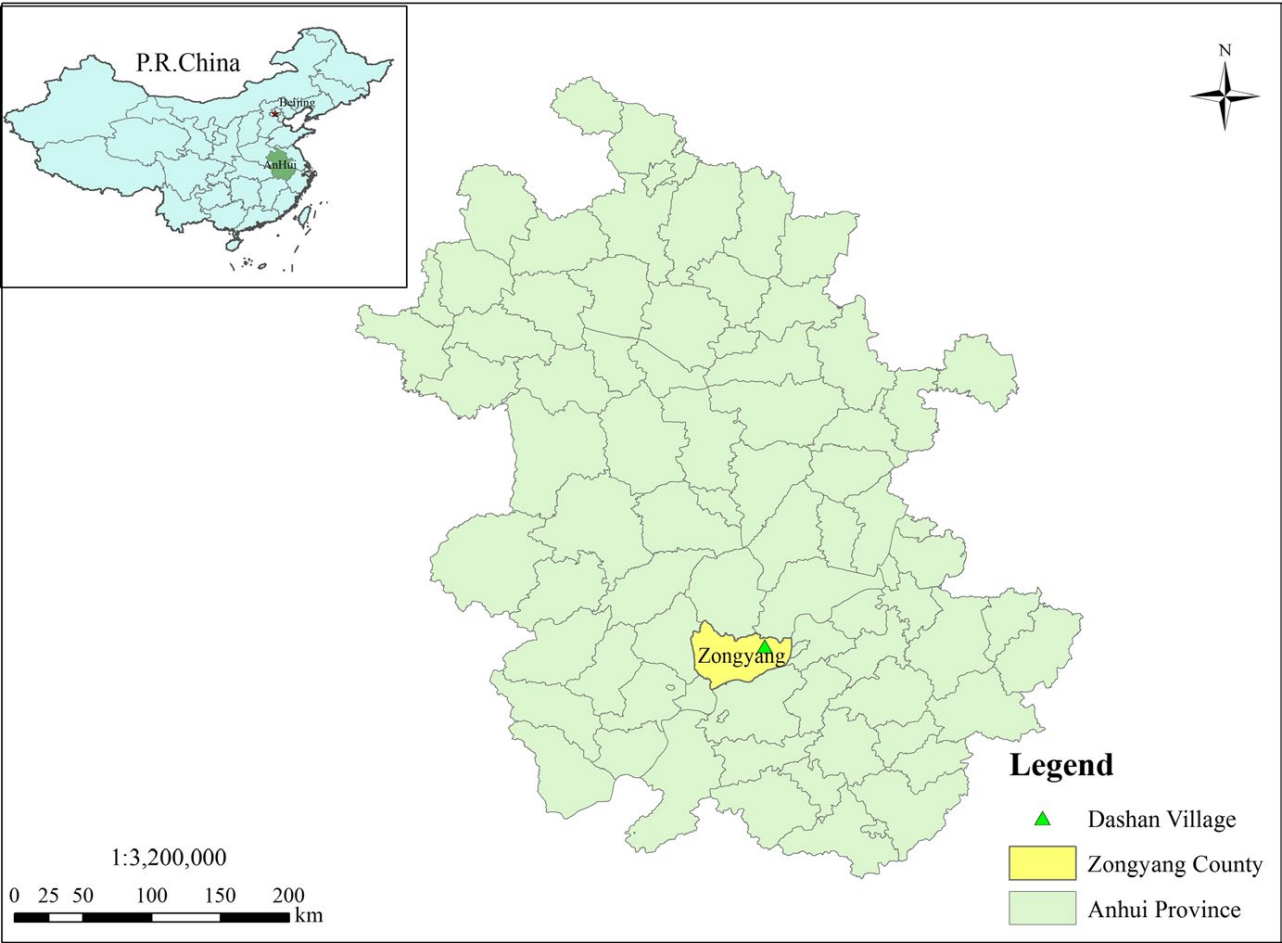
Location of the study area (Dashan Village, Zongyang County, Anhui Province, China)

This study was conducted in the subtropical mountain area and located in Dashan Village, Zongyang County, Anhui Province, Southeastern China (31°01′–31°38′N, 117°05′–117°43′E, and 160 m above sea level). Zongyang County was situated by the edge of southeast of the Dabie Mountains, and it located at the north shore of the Yangtze River in the Midwest area of Anhui province (see Figure 1). It belongs to subtropical monsoon humid climate zone with typical northern subtropical hilly land climate characteristics. The mean annual temperature in this area was 16.5°C, the mean annual sunshine hours to 2,064.9 hr and the mean annual precipitation is 1,326.5 mm of which 60% to 70% mainly distributes in June, July, August, and September. The soil of the study site was classified as yellow brown granite with lighter texture and the pH value was 5.5–6.5.

**Figure 2:**
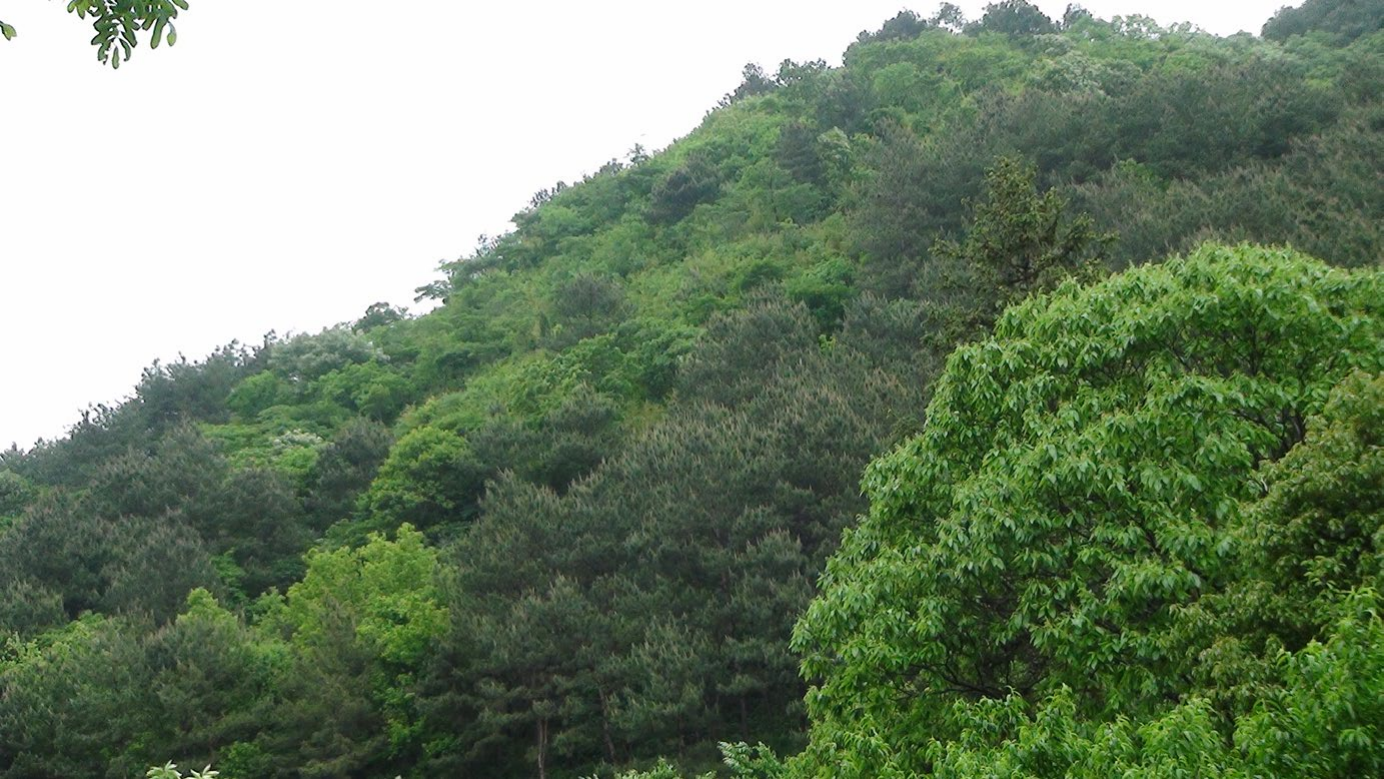
Study area of *Pinus massoniana* mixed forest

The topographical feature in the study area was low hilly land with gentle slope and the slopes were usually more than 20 degrees, and part of them up to 30 degrees. The coniferous and broad‐leaved mixed forests were distributed in natural forest of the forest edge in Dashan Village, Zongyang County, and most of its forests were the artificial plantations of *Pinus massoniana* forest with not tending. After 40 years of closing hillsides to facilitate afforestation, some broad‐leaved tree species invaded and the forest developed into a needle, broad‐leaved mixed forests in this region, so this kind of coniferous and broad‐leaved mixed forest types were transition from the artificial *Pinus* forest to seminatural broad‐leaved forest. Evergreen and deciduous broad‐leaved mixed forest of *Pinus massoniana* was the main vegetation types (including the *P. massoniana*–*Q. tissima* mixed forest [PQM], the *P. massoniana*–*L. formosana* mixed forest [PLM] and the *P. massoniana*–*P. strobilacea* mixed forest [PPM]) in this study area (Figure 2). There was a clear forest community structure and the canopy density from 0.6 to 0.8.

### 2.2 Sampling design

The sampling and sample determination were conducted in Mid‐August of 2014. Four forest types (PF, PQM, PLM, and PPM) were chosen to take samples. For each forest type, three sample plots with 20 × 20 m were set to collect the plant leaf samples. More detailed information for the four *Pinus massoniana* forest types were shown in Table 1.

**Table 1:** General situation of four *Pinus massoniana* forest types in a subtropical mountain, Southeastern China

Forest type	Proportion of mixture	Density (each plant per hm^2^)	Average height/m	Average DBH/cm	Altitude/m	Slope aspect	Slope gradient/(°)	Slope position	Arbor cover/%	Dominant species in tree layer
PF	–	1,510	9.2	11.9	105	Northwest	23	Medium	60	*P. massoniana*
					99	North	18	Medium	60	
					85	North	20	Down	70	
PQM	2:1	1,120 (450)	10.3 (8.9)	14.1 (9.8)	90	North	15	Down	70	*P. massoniana*, *Q. tissima*, *P. strobilacea*
					115	Northwest	18	Medium	80	
					138	North	20	Up	70	
PLM	2:1	1,050 (515)	10.5 (12.9)	13.6 (11.7)	142	Northwest	22	Up	65	*P. massoniana*, *P. strobilacea*, *L. formosana*
					153	Northwest	20	Up	70	
					118	North	18	Medium	75	
PPM	2:1	950 (460)	9.8 (10.2)	12.8 (10.5)	110	North	20	Medium	70	*P. massoniana*, *P. strobilacea*, *L. formosana*
					95	Northwest	16	Down	65	
					98	Northwest	18	Down	70	

Note

The data in bracket is *Q. tissima*,* L. formosana*, or *P. strobilacea*, the same below.

Abbreviations: PF: Pure *Pinus massoniana* forest; PLM: *Pinus massoniana* and *Liquidambar formosana* mixed forest; PPM: *Pinus massoniana* and *Platycarya strobilacea* mixed forest; PQM: *Pinus massoniana* and *Quercusacu tissima* mixed forest.

### 2.3 Plant leaf functional traits

On Mid‐August, six plants of each tree species (*P. massoniana*, *Q. tissima*,* L. formosana*, and *P. strobilacea*) at each plot were chosen, then 8–10 mature fully expanded leaves were picked as a sample and totally 48–60 leaves from each species were measured in each plot.

Leaf area (LA) was determined by scanning the leaves with a flatbed scanner and analyzing the images using an area measurement software by Motic (Motic images advanced 3.0, China). After the leaf area and leaf saturated fresh mass were measured, all leaf samples were carefully put into paper bags and then oven‐dried at 70°C for at least 48 hr to a constant mass and measured to determine their dry mass. The leaf area and specific leaf area (SLA) reported for each plant represents the average obtained from the 8–10 leaves per individual collected at each canopy position. The SLA (cm^2^/g) was computed as the ratio of leaf area (LA) to leaf dry mass (LM), and leaf dry matter content (LDMC) was computed as the ratio of leaf dry mass to leaf saturated fresh mass, respectively.

After the determination of SLA, the dried leaf samples were used for leaf nitrogen, phosphorus and carbon content determination. All the leaves from the same species at the same sites were mixed into a single sample and grounded to 100‐mesh for chemical determination. Leaf C and N content were analyzed using a Elementar analyzer (Elementar, Vario pyro Analyzer, Mettler‐Toledo International Inc.), and the leaf N content was expressed on a mass basis (N_mass_, %). Total leaf P concentrations were measured by a molybdate/stannous chloride method (Kuo, 1996) after H_2_SO_4_–H_2_O_2_–HF digestion (Bowman, 1988) quantified by reference to a national standard material with a known P concentration (UV2700 spectrophotometer, Japan). All the chemical determinations were repeated three times with the same samples and express on mass basis (%). Mass ratios of C:N, C:P, and N:P were used here to facilitate the comparison with the terrestrial ecology literature (Güsewell, 2004; Table 2).

**Table 2:** Definitions of abbreviations, acronyms, and units of leaf functional traits

Abbreviation	Definition	Units	Formula/instrument/software
SLA	Specific leaf area	cm^2^/g	Leaf area/leaf dry mass
LDMC	Leaf dry matter content	g/g	Leaf dry mass/leaf saturated fresh mass
LM	Leaf dry mass	g	Weigh
LA	Leaf area	cm^2^	Motic images advanced 3.0
LCC	Leaf carbon concentration	%	Elementar analyzer
LNC	Leaf nitrogen concentration	%	Elementar analyzer
LPC	Leaf phosphorus concentration	%	Molybdate/stannous chloride method
C/N	Leaf carbon/nitrogen ratio	–	C/N = LCC/LNC
C/P	Leaf carbon/phosphorus ratio	–	C/P = LCC/LPC
N/P	Leaf nitrogen/phosphorus ratio	–	N/P = LNC/LPC

### 2.4 Data analysis

To examine the effect of forest types on plant leaf functional traits for each species, we conducted One‐way analysis of variance (ANOVAs) using forest types as the predictor. Significant differences were evaluated at the *P < *0.05 level. When significance was observed, the LSD (least significant difference) post hoc test was used to conduct multiple comparisons of plant leaf functional traits among treatments. The Pearson correlation analysis was used to test the relationships between leaf functional traits (SLA, LDMC, LA, LM and LNC, LPC, LCC, N/P, C/N, C/P). All data were expressed as the mean ± standard deviation (*SD*). All statistical analyses were performed using SPSS 17.0 software (SPSS Inc.).

## 3 RESULTS

### 3.1 Effects of forest types on leaf morphological traits

All the leaf samples of four *P. massoniana* forest types taken in the subtropical mountain, SLA and LA exhibited large variations, SLA ranging from 45.31 to 286.15 cm^2^/g and LA from 1.38 to 38.29 cm^2^, and their means were 135.09 cm^2^/g and 16.60 cm^2^, respectively. LDMC and LM exhibited small variations, LDMC ranging from 0.30–0.44 g/g and LM from 0.02 to 0.15 g, respectively (Table 3).

**Table 3:** Changes in leaf morphological traits among the four *Pinus massoniana* forest types in the subtropical mountain, Southeastern China (mean ± *SD*)

Forest type	Species	SLA	LDMC	LM	LA
PF	*P. massoniana*	*46.80* ± 1.18aC	*0.44* ± 0.02aA	*0.041* ± 0.003aC	*1.91* ± 0.09aB
PQM	*P. massoniana*	*53.98* ± 2.12a	*0.37* ± 0.01b	*0.034* ± 0.003a	*1.48* ± 0.39a
	*Q. tissima*	*232.31* ± 3.65B	*0.33* ± 0.01B	*0.125* ± 0.01B	*36.22* ± 2.41A
	Mean	*143.15* ± 126.10	*0.35* ± 0.03	*0.080* ± 0.01	*18.85* ± 24.56
PLM	*P. massoniana*	*55.31* ± 2.20a	*0.35* ± 0.02b	*0.027* ± 0.002a	*1.58* ± 0.48a
	*L. formosana*	*286.15* ± 9.24A	*0.30* ± 0.03B	*0.120* ± 0.01B	*38.29* ± 3.16A
	Mean	*170.73* ± 163.23	*0.33* ± 0.04	*0.074* ± 0.01	*19.94* ± 25.96
PPM	*P. massoniana*	*45.31* ± 1.67a	*0.38* ± 0.02b	*0.037* ± 0.001a	*1.38* ± 0.22a
	*P. strobilacea*	*225.79* ± 8.48B	*0.34* ± 0.01B	*0.144* ± 0.01A	*35.33* ± 3.13A
	Mean	*135.56* ± 127.62	*0.36* ± 0.03	*0.091* ± 0.01	*18.36* ± 24.01
	Significance	*p* > 0.05, *p* < 0.05	*p* < 0.05, *p* < 0.05	*p* > 0.05, *p* < 0.05	*p* > 0.05, *p* < 0.05

Note

Different small letters in the same column indicated significant difference among the species of *P. massoniana* in the four forest types, and different capital letters indicated significant difference among different plant species in the four types at 0.05 level, the same below.

The italic values represent the Latin names of the four species among the four forest types.

**Figure 3:**
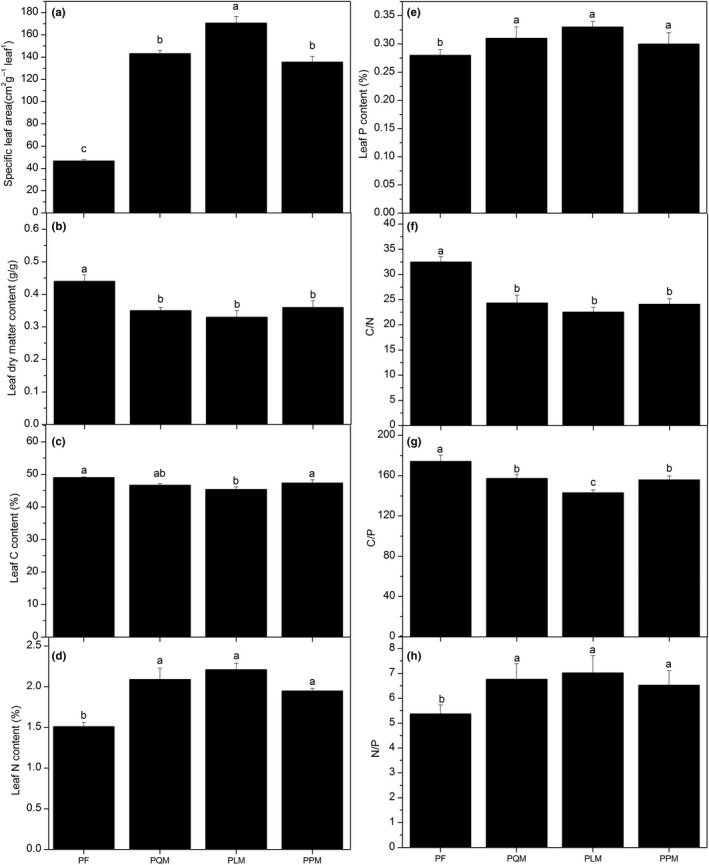
Leaf morphological traits, nutrients, and stoichiometric traits among the four *P. massoniana* forest types in the subtropical mountain, Southeastern China. The data are in the form of mean ± *SD*. Bars with the different letters above them mean significant differences at *p* < 0.05. In the horizontal axis, PF, Pure *P. massoniana* forest (*n* = 1 species); PQM, *P. massoniana*, and *Q. tissima* mixed forest (*n* = 2 species); PLM, *P. massoniana*, and *L. formosana* mixed forest (*n* = 2 species); PPM, *P. massoniana*, and *P. strobilace* mixed forest (*n* = 2 species)

SLA, LM, and LA of *P. massoniana* were not differed significantly (*p* > 0.05) among the four forest types, except for LDMC (Table 3). Comparatively, SLA, LM, and LA were significantly higher in the three broad‐leaved species (*Q. tissima*,* L. formosana*, and* P. strobilacea*) than in the coniferous species of *P. massoniana* (*p* < 0.05), especially SLA of *L. formosana* in PLM type (Figure 3a). Significant difference was observed in LDMC between coniferous species and the three broad‐leaved species (Table 3, Figure 3b, *p* < 0.05).

### 3.2 Effects of forest types on leaf nutrients and stoichiometric traits

For all species, the range of variation of LCC, LNC, LPC, C:N, C:P, and N:P ratios ranged from 44.27% to 49.04%, 1.5% to 2.8%, 0.2% to 0.4%, 16.22 to 32.51, 129.12 to 174.3, and 5.3 to 8.1 in the four forest types, with a mean of 46.83%, 2.02%, 0.31%, 24.92, 155.37, and 6.56, respectively (Table 4).

**Table 4:** Changes in leaf nutrients and stoichiometric traits among the four *Pinus massoniana* forest types in the subtropical mountain, Southeastern China (mean ± *SD*)

Forest type	Species	LCC (%)	LNC (%)	LPC(%)	C/N	C/P	N/P
PF	*P. massoniana*	*49.04* ± 0.14aA	*1.51* ± 0.05aB	*0.28* ± 0.01aB	*32.51* ± 1.04aA	*174.30* ± 6.18aA	*5.37* ± 0.36aB
PQM	*P. massoniana*	*46.50* ± 0.31b	*1.59* ± 0.08a	*0.28* ± 0.01a	*30.58* ± 1.58a	*171.99* ± 0.64a	*5.63* ± 0.45a
	*Q. tissima*	*46.86* ± 0.61AB	*2.59* ± 0.20A	*0.33* ± 0.02A	*18.09* ± 1.45B	*143.11* ± 6.90B	*7.91* ± 0.79A
	Mean	*46.68* ± 0.25	*2.09* ± 0.71	*0.31* ± 0.04	*24.34* ± 8.83	*157.55* ± 20.42	*6.77* ± 1.61
PLM	*P. massoniana*	*46.50* ± 1.08b	*1.64* ± 0.06a	*0.30* ± 0.01a	*28.84* ± 0.94a	*157.03* ± 3.80b	*5.99* ± 0.66a
	*L. formosana*	*44.27* ± 0.37B	*2.78* ± 0.09A	*0.35* ± 0.01A	*16.22* ± 0.92B	*129.12* ± 2.32C	*8.04* ± 0.71A
	Mean	*45.39* ± 1.58	*2.21* ± 0.81	*0.33* ± 0.04	*22.53* ± 8.92	*143.08* ± 19.74	*7.02* ± 1.45
PPM	*P. massoniana*	*47.00* ± 2.03b	*1.52* ± 0.03a	*0.28* ± 0.02a	*28.97* ± 2.10a	*169.76* ± 5.71a	*5.56* ± 0.95a
	*P. strobilacea*	*47.65* ± 0.03A	*2.38* ± 0.02A	*0.32* ± 0.01A B	*19.22* ± 0.12B	*142.31* ± 1.60B	*7.41* ± 0.21A
	Mean	*47.33* ± 0.46	*1.95* ± 0.61	*0.30* ± 0.03	*24.10* ± 6.89	*156.04* ± 19.41	*6.53* ± 1.31
	Significance	*p* < 0.05	*p* < 0.05	*p* < 0.05	*p* < 0.05	*p* < 0.05	*p* < 0.05

Note

The italic values represent the Latin names of the four species among the four forest types.

Leaf C, N, and P contents varied significantly (Figure 3c–e, *p* < 0.05) among the four forest types (Table 4). In details, there was significant difference in LCC of *P. massoniana*, but no differences in LNC and LPC of *P. massoniana* among the four forest types. *P. massoniana* had the highest leaf C content in the PF forest type and significant higher than *L. formosana* in the PLM forest type (*p* < 0.05). However, the deciduous trees had significantly higher leaf N and P contents than the evergreen tree—*P. massoniana*, and the values were in the order of *L. formosana* (PLM) > *Q. tissima* (PQM) > *P. strobilacea* (PPM) > *P. massoniana* (PF) (Figure 3d,e). LNC and LPC of *P. massoniana* did not show significant changes among the four forest types (*p* > 0.05).

C:N, C:P, and N:P ratios of *P. massoniana* showed no differences among the four forest types (*p* > 0.05), except for *P. massoniana* in the PLM forest type. C:N and C:P ratios of *P. massoniana* in the PF forest type was significantly higher than the three deciduous trees in mixed forests, while N:P ratio of *P. massoniana* was significantly lower than the three deciduous trees (*p* < 0.05; Table 4, Figure 3f–h). Comparatively, the three deciduous trees had higher N, P, N:P ratio and lower C, C:N, C:P ratios than the evergreen tree—*P. massoniana*, especially in the PLM forest types.

### 3.3 Relationships among leaf functional traits

Correlation patterns among leaf functional traits in four forest types were observed (Table 5). Leaf functional traits were not independent of each other. Leaf morphological traits showed a significant negative correlation with LDMC and SLA (*R* ^2 ^= −0.713, *p* < 0.05). LDMC was also significantly and negatively correlated with LA (*R* ^2 ^= −0.660, *p* < 0.05). Leaf morphological traits were also correlated significantly with leaf nutrients traits. Across all species, SLA was positively correlated with LNC (*R* ^2 ^= 0.947, *p* < 0.01), LPC (*R* ^2 ^= 0.806, *p* < 0.01) and negatively correlated with LCC (*R* ^2 ^= −0.716, *p* < 0.05). However, LDMC was positively correlated with LCC, but negatively correlated with LNC and LPC (*p* < 0.05, Table 5).

**Table 5:** Correlation coefficients among leaf functional traits among the four *Pinus massoniana* forest types in the subtropical mountain, Southeastern China

Parameters	SLA	LDMC	LM	LA	LCC	LNC	LPC
SLA	1.000						
LDMC	−0.713*	1.000					
LM	–	–	1.000				
LA	–	−0.660*	0.848*	1.000			
LCC	−0.716*	0.711*	−0.661	−0.464	1.000		
LNC	0.947**	−0.703*	−0.563	0.433	−0.675*	1.000	
LPC	0.806**	−0.630*	−0.365	0.286	−0.543	0.878*	1.000
C/N	−0.544	0.759*	−0.882	−0.532	–	–	−0.838*
N/P	0.518	−0.557	0.891	0.515	−0.672*	–	–
C/P	−0.483	0.721*	−0.776*	−0.457	–	−0.915*	–

Note

“–”indicates that autocorrelation exists and no analysis is conducted.

* The correlation is significant at *p* = 0.05 (2‐tailed).

** The correlation is significant at *p* = 0.0l (2‐tailed).

With regard to the relationships among the leaf nutrient traits, LCC was negatively correlated with leaf N and P, and the correlation between LCC and LNC was the strongest (*R* ^2 ^= −0.675, *p* < 0.05). However, leaf N and P were significantly and positively correlated with each another (*R* ^2 ^= 0.878, *p* < 0.01). LCC was negatively correlated with N:P ratio (*R* ^2 ^= −0.672, *p* < 0.05), LNC and LPC were also negatively correlated with C:P (*R* ^2 ^= −0.915, *p* < 0.01) and C:N ratios (*R* ^2 ^= −0.838, *p* < 0.01). This indicated that there was a trade‐off of C, N, and P nutrient allocation in plant fast growth, and which also showed N and P were coordinated elements.

## 4 DISCUSSION

Leaves directly support plant growth by converting light and carbon dioxide into chemical energy via photosynthesis (Cochrane et al., 2016). Specific leaf area (SLA) is closely correlated with photosynthetic capacity and leaf nitrogen content and is important for growth (Wright et al., 2004). The analysis of our study indicated that the deciduous species of *L. formosana* exhibited the highest SLA in PLM forest type, and significantly higher than the other three species (*p* < 0.05, Table 3), while the SLA in the evergreen species *P. massoniana* were not differed significantly (*p* > 0.05) among the four forest types. The values were in the order of *L. formosana* (PLM) > *Q. tissima* (PQM) > *P. strobilacea* (PPM) > *P. massoniana* (PF) (Figure 3a). Regarding the results from correlation analyses among leaf functional traits, we found a significantly positive correlation between SLA and LNC (the correlation coefficient was 0.947, *p* < 0.01, Table 5), indicating species of *L. formosana* exhibited high SLA and LNC, as well as a higher leaf area (LA) and thin leaves (low LM) in the PLM mixed forest type (Table 3).

Many studies have shown that deciduous species were characterized by trait values generally associated with productivity, namely high leaf nitrogen and thin leaves (high SLA and low LM), indicating high photosynthetic capacities and high growth rates. In contrast, evergreen species were characterized by trait values associated with persistence and slow growth, namely thick (high LM), nitrogen‐poor leaves (low SLA and LNC; Li et al., 2017; Wang, Zhou, Xiao, Liu, & Wang, 2017). Some researchers have found leaf thickness to be a very valuable feature, efficiently enabling resource acquisition, water saving and CO_2_ assimilation, and studies have detected that SLA decreased and leaf thickness increased during leaf phylogenetic development (Costa‐Saura et al., 2016; Cornelissen et al., 2003). Usually, species with low SLA values as a decrease in leaf surface area would mean fewer avenues for water loss and are focused on the conservation of acquired resources, due to their large dry mass and leaf dry matter content, high concentrations of cell walls and secondary metabolites (Laureano et al., 2008). However, fast‐growing deciduous species invested less in the production of nonphotosynthetic leaf tissues and formed larger, lighter leaves with higher SLA, and consequently exhibit a higher leaf N and P content, compared with evergreen species (Ivanova et al., 2018; Wright et al., 2004). These plant morphological traits have a close relationship with plant water use efficiency, and the ability to acquire nutrient resources and are thus better adapted to different environments. The strategies reflected by these trait syndromes appear fairy universal and are now conceptualized in the leaf economics spectrum (Poorter & Bongers, 2006).

In this study, evergreen species of *P.massoniana* had the highest LDMC and LCC (0.44 g/g and 49.04%, respectively) in the PF forest type than the other three mixed forest types (PQM, PLM, and PPM) (*p* < 0.05, Tables 3 and 4, Figure 3b,c) and also had a significantly negative correlation with SLA (the correlation coefficient was 0.713, *p* < 0.05, Table 5). Previous studies have shown that leaves of evergreen plants usually have longer lifespan, higher leaf C content, and higher costs of construction and maintenance than leaves of deciduous species (Chaturvedi, Raghubanshi, & Singh, 2011; Osnas, Lichstein, Reich, & Pacala, 2013), and this indicate lower photosynthetic rates and comparatively slower growth rate with strong defense capabilities. Construction costs are the sum of all the carbon and energy utilized in producing a net gain in dry weight, therefore species with high LDMC tend to invest more dry matter per leaf in a nutrient‐poor environment and can also likely prevent water loss from the leaf surface more effectively (Suter & Edwards, 2013). In contrast, three kinds of deciduous species (*Q. tissima*, *L. formosana*, and *P. strobilace*) in the mixed forest types had significant higher leaf N and P content than the evergreen species of *P. massoniana* in the PF forest type (Table 4, Figure 3d,e). Studies found that species on the “fast return” (rapid growth, thin leaves, high nutrient concentrations, and high rates of photosynthesis) decompose more quickly than species on the “slow return” (slow growth, thicker, more recalcitrant leaves with more defenses and lower rates of photosynthesis), suggesting that the suite of coordinated structural and chemical leaf traits maximizing photosynthesis also has important implications for nutrient cycling (Funk et al., 2016). So, compared with the pure *P. massoniana* forest, *P. massoniana* mixed forest has more advantages in improving leaf nutrient content by adding deciduous species.

The average leaf C content across all species among the four forest types were 46.83%, significantly higher than that in the other regions of China (in the Loess Plateau [43.8%] and Mu Us Sandy Land [41.66%] located in the northwest part of China; Elser et al., 2000), which shows the higher leaf organic compound contents of species in the subtropical mountain of Southeastern China (Table 6). Leaf N content of the plants in the subtropical mountain was 2.02%, significantly lower than that in the Loess Plateau of China (2.41%) and in the South Texas of USA (2.39%) (Qin, Shangguan, & Xi, 2018), and equal to other regions in the word (2.06% and 2.01%, respectively; Elser et al., 2000; Reich & Oleksyn, 2004). The average leaf P content was 0.31%, and much higher than that of reported in other regions in China and in the world (Table 6). Compared with N, it is suggested that the plants are more rich in P in the South of China.

**Table 6:** Comparison of leaf nutrient traits between the subtropical mountain and other regions

Study areas	C (%)	N (%)	P (%)	C/N	C/P	N/P	References
Subtropical mountain, Anhui	46.83 ± 1.44 (*n* = 7)	2.02 ± 0.56 (*n* = 7)	0.31 ± 0.03 (*n* = 7)	24.92 ± 6.79 (*n* = 7)	155.37 ± 17.58 (*n* = 7)	6.56 ± 1.18 (*n* = 7)	This study
Loess Plateau	43.80 ± 4.30 (*n* = 126)	2.41 ± 0.85 (*n* = 126)	0.160 ± 0.06 (*n* = 126)	21.2 ± 10.2 (*n* = 126)	312 ± 13.5 (*n* = 126)	15.4 ± 3.9 (*n* = 126)	Zheng and Shangguan (2007)
Mu Us Sandy Land	41.66 ± 5.33 (*n* = 30)	2.04 ± 0.11 (*n* = 30)	–	23.32 ± 2.02 (*n* = 30)	–	–	Zhu et al. (2015)
China	–	2.02 ± 0.84 (*n* = 554)	0.146 ± 0.10 (*n* = 745)	–	–	16.3 ± 9.32 (*n* = 547)	Han, Fang, Guo, and Zhang (2005)
South Texas, USA	46.80 ± 2.79 (*n* = 4)	2.39 ± 0.49 (*n* = 4)	0.15 ± 0.04 (*n* = 4)	21.01 ± 5.36 (*n* = 4)	345.07 ± 111.43 (*n* = 4)	17.35 ± 2.04 (*n* = 4)	Qin et al. (2018)
Global	–	2.01 ± 0.87 (*n* = 1,251)	0.177 ± 0.11 (*n* = 923)	–	–	13.8 ± 9.47 (*n* = 894)	Reich and Oleksyn (2004)
Global	46.40 ± 3.21 (*n* = 492)	2.06 ± 1.22 (*n* = 398)	0.199 ± 0.15 (*n* = 406)	22.5 ± 10.6 (*n* = 398)	232 ± 145 (*n* = 406)	12.7 ± 6.82 (*n* = 325)	Elser et al. (2000)

Leaf C:N and C:P ratios reflect carbon assimilation capacity, and to a certain extent, the efficiency of nutrient utilization which has an important ecological significance (Sterner & Elser, 2002). The average C:N and C:P ratios of all the species in the four forest types were 24.92 and 155.37, respectively, and have higher C/N ratio but significantly lower C/P ratio than those in other regions in China and global vegetation (22.5 and 232, respectively), it indicates that the absorption capacity and utilization efficiency of N is higher than that of P in this study area (Elser et al., 2000).

Of various mineral elements, N and P are generally considered the major growth‐constraining nutrients in plant communities worldwide, and leaf N:P ratio has been used to detect conditions of N or P limitations (Garrish, Cernusak, Winter, & Turner, 2010). Usually, an N/P ratio less than 14 generally indicates N constraint, while an N/P ratio more than 16 suggests P constraint. N/P ratio within 14 and 16, either N or P or both of them constrain plant growth (Garrish et al., 2010). In our study area, Leaf N/P ratio was 6.56, significantly lower than Chinese average of 16 and global levels of 13 (Table 6). Thus, it is also suggested that plant growth in the subtropical mountain is liable to be constrained by N, and this is mainly due to the effects of acidic soil types in south of China. Because of the high temperature and rainfall in south China, soil acidification is easy to occur in this region, so soil phosphorus content is abundant but nitrogen nutrient is easily leached in this kind of soil type.

Among the species in this study in the subtropical mountain, leaf C content was negatively correlated with leaf N and P content (−0.675 [*p* < 0.05] and −0.543, Table 5), and this indicated that there was a trade‐off in nutrient allocation between structural toughness and fast growth. And the correlation between leaf N and leaf P content was the strongest in our studied forest community (0.878, *p* < 0.01, Table 5), which is consistent with the results from global compilations of data across different ecosystem types and plant functional groups, including herbaceous and woody species (Li et al., 2017; Qin et al., 2016; Riva et al., 2016). Because of the highly positive correlations between leaf N and P, N/P ratios of different forest types varied less than N or P alone, except for the differences between broad‐leaved and coniferous species. The relatively stable N/P ratios of forest types may reflect a fundamental feature of plant with respect to leaf N and P stoichiometry. And this strong correlation between N and P concentrations may result from the most basic biochemical processes—metabolic activities shared by terrestrial plants, such as photosynthesis and respiration (Marschner, 2012).

Our analyses of the patterns of variation in leaf functional traits across the studied woody species in a subtropical forest in China showed that leaf trait coordination was associated with forest types and additionally with different plant species. According to the results of our study, for the two kinds of species, the evergreen conifer species of *P. massoniana* had higher LDMC, leaf C content, C/N and C/P ratios. This indicates that *P. massoniana* has adapted to the nutrient‐poor environment by increasing their leaf dry matter for higher construction costs thereby reducing water loss and reflects a resource conservation strategy in this study area. While the three kinds of deciduous broad‐leaved species had higher SLA, leaf N, leaf P nutrient contents, and N/P ratio, showing the three deciduous species of *L. formosana*, *Q. tissima*, and* P. strobilacea* exhibited an optimized resource acquisition strategy rather than resource conservation strategy. For the four forest types, the three mixed forest types exhibited advantages in leaf nutrient increases more than the pure *P. massoniana* forest (PF), especially PLM forest type, and its order was as follows: PLM > PQM > PPM (Figure 3). Thus, the differences in four species regarding plant leaf traits were greatly affected by the forest types and further suggest that studying leaf functional traits at regional scales are more and more important for accurately understanding vegetation–environment relationships at a global scale. Therefore, unraveling history and functional background of leaf traits has the potential to substantially improve our understanding of plant evolution and its interrelationship with environment (Roth‐Nebelsick et al., 2017).

## CONFLICT OF INTEREST

None declared.

## AUTHORS' CONTRIBUTIONS

J. Qin designed this study and conducted the experiment. J. Qin wrote the paper. Z.P. ShangGuan revised the paper. All authors provided editorial advice and approved the final version.

## Data Availability

All data generated or analyzed in this study are included in this published article.
