#  

**DOI:** 10.1002/ece3.8728

**Published:** 2022-03-21

In the article by Qin and Shangguan (2019), the authors would like to correct the two occurrences of the grant number from “KJ2019A” to “KJ2019A0206” in the Funding Information and Acknowledgments section.

It should have read as follows:

**Funding information**

The Natural Science Research Key Project of Universities in Anhui Province “Study on community assembly of Pinus massoniana evergreen deciduous and broad‐leaved mixed forests in the northern subtropical based on plant functional traits”, Grant/ Award Number: KJ2019A0206; Natural Science Foundation of Anhui Province, Grant/Award Number: 1408085QC57; the Strategic Priority Research Program of the Chinese Academy of Sciences (XDA23070201); The Outstanding Young Talent Foundation of Anhui Province, Grant/Award Number: 2012SQRL058; The Youth Science Foundation of Anhui Agricultural University, Grant/Award Number: 2012zd015.

**Acknowledgments**

This work was supported by the Natural Science Research Key Project of Universities in Anhui Province “Study on community assembly of Pinus massoniana evergreen deciduous and broad‐leaved mixed forests in the northern subtropical based on plant functional traits” (KJ2019A02060), Natural Science Foundation of Anhui Province (1408085QC57), the Strategic Priority Research Program of the Chinese Academy of Sciences (XDA23070201), the Outstanding Young Talent Foundation of Anhui Province (2012SQRL058), and the Youth Science Foundation of Anhui Agricultural University (2012zd015). The authors express their sincere thanks to the reviewers and issue editors of the journal for their valuable comments, suggestions, and revisions to this manuscript.

The authors would like to apologize for this error.
